# Design of a Hyper-Redundant Robot and Teleoperation Using Mixed Reality for Inspection Tasks

**DOI:** 10.3390/s20082181

**Published:** 2020-04-12

**Authors:** Andrés Martín-Barrio, Juan Jesús Roldán-Gómez, Iván Rodríguez, Jaime del Cerro, Antonio Barrientos

**Affiliations:** Centre for Automation and Robotics (UPM-CSIC), José Gutiérrez Abascal, 2, 28006 Madrid, Spain

**Keywords:** hyper-redundant, robot, design, teleoperation, mixed reality, inspection, Industry 4.0

## Abstract

Hyper-redundant robots are highly articulated devices that present numerous technical challenges such as their design, control or remote operation. However, they offer superior kinematic skills than traditional robots for multiple applications. This work proposes an original and custom-made design for a discrete and hyper-redundant manipulator. It is comprised of 7 sections actuated by cables and 14 degrees of freedom. It has been optimized to be very robust, accurate and capable of moving payloads with high dexterity. Furthermore, it has been efficiently controlled from the actuators to high-level strategies based on the management of its shape. However, these highly articulated systems often exhibit complex shapes that frustrate their spatial understanding. Immersive technologies emerge as a good solution to remotely and safely teleoperate the presented robot for an inspection task in a hazardous environment. Experimental results validate the proposed robot design and control strategies. As a result, it is concluded that hyper-redundant robots and immersive technologies should play an important role in the near future of automated and remote applications.

## 1. Introduction

Early robots were initially designed for manufacturing purposes in industry. They were devised to perform repetitive tasks in highly controlled environments [1]. Traditional serial manipulators were expected to position and orient their end-effector in the three-dimensional (3D) space for different applications. To do so, a robot needs to have a minimum number of 6 independent movements, also called Degrees of Freedom (DoF) [2].

At the same time, robots with more than 6 degrees of freedom were devised. Such manipulators exhibited dexterous kinematic skills, fault-tolerance and higher capabilities to move in unstructured environments. Because of their large number of degrees of freedom, they were called hyper-redundant robots. Initially, these devices were discrete and made by rigid materials as a logical evolution of traditional ones. However, as time progressed, other variants composed by flexible materials were proposed, so they could continuously bend instead of having distinguishable links. These mechanisms were called continuous robots. Recently, last research efforts have taken a step further creating robots made by soft materials, capable of adapting to their environment and being inherently safe to interact with their surroundings [3].

However, the evolution of discrete hyper-redundant robots to flexible or soft structures does not entail a replacement of the first by the second ones. Thus, they are devised for different applications. For example, continuous and soft robots have higher adaptability skills and are safer to interact with their environment. However, they are usually less sturdy, accurate and struggle to carry heavy payloads compared to rigid manipulators. Thus, discrete and rigid hyper-redundant robots are better suited for applications in hazardous or adverse environments such as for inspection tasks in hazardous environments. On the other hand, continuous and soft robots are more compliant and exhibit better capabilities to surround delicate objects like fruit or vegetables [4].

Once a hyper-redundant robot is designed, it is necessary to extract its behavior using a reliable and accurate model. This model is expected to describe the robot behavior under the influence of an actuation system, so their movements can be predicted beforehand. Modelling discrete hyper-redundant robots is a relatively simple task because different values in the actuators can be linearly interpolated to obtain displacements and/or rotations in the joints between links. However, continuous and soft robots usually require complex modelling techniques to describe and manage their numerous non-linearities [5]. The latest research also proposes model-free approaches relying on large datasets that collect experimental data from the robot and then used with machine learning algorithms to extract their behavior [6].

Then, if the behavior of the robot is reliable and well described, different control strategies need to be developed to adequately manage their movements. It is possible to distinguish 3 different levels of control. (i) A low-level control based on managing appropriate inputs for the actuators. (ii) When the actuation is not located in the robot body, a mid-level control should study how to transfer the movements from the actuators to the joints. (iii) And high-level controls that rely on previous ones to manage the whole-body movements from a higher level of abstraction such as solving the inverse kinematics problem [7], guiding the robot with a follow-the-leader strategy [8], or performing shape controls [9], among many others.

Additionally, since hyper-redundant robots have many degrees of freedom, they often exhibit complex shapes that may frustrate their spatial understanding. Thus, controlling the pose of such robots may be difficult using conventional tools. Strategies to adequately define such references are usually studied in *telerobotics*. This discipline links a human operator and a robot to execute a remote task. If the information about the remote environment is naturally displayed to the operator, the application will have a high level of telepresence [10]. Since hyper-redundant robots often exhibit complex shapes in the 3D space, maybe immersive technologies can be a good solution to solve this problem [11].

Immersive realities can be understood as those real or simulated environments in which a perceiver experiences telepresence [12,13]. They can be classified in 3 categories: (i) Virtual Reality (VR) provides interaction of virtual elements in virtual environments; (ii) Augmented Reality (AR) features interaction of virtual elements in real environments; and (iii) Mixed Reality (MR) offers interaction of real and virtual elements through real or virtual environments [14]. Immersive realities are considered one of the main technologies in Industry 4.0. This revolution aims to combine all the production agents in the shape of cyber-physical systems by means of network connections and information management [15]. Immersive technologies will allow multiple applications such as remote and virtual assistance, augmented reality manuals, indoor guidance, training of operators, immersive 3D design or even teleoperation of robots in remote environments (Section 5).

This work presents an original design of a discrete hyper-redundant robot, optimized to be very sturdy, accurate and capable of moving reasonably heavy payloads for inspection purposes. Furthermore, this work proposes an original method to teleoperate this kind of robots using Mixed Reality. Thus, the hypothesis of this work states that hyper-redundant robots, like the one presented, can be a good solution to perform complex tasks in remote environments. This assumption will be further analyzed throughout this document.

The following Section presents a summary of relevant previous works; Section 3 explains the design process of the robot from a mechanical, actuation and electronic perspective; Section 4 proposes a kinematic scheme to control its movements from the actuators to a high-level control; Section 5 introduces a real application where the robot is teleoperated using Mixed Reality for an inspection task; and finally, Section 6 outlines the conclusions derived from this work.

## 2. State of the Art

Robots have become popular as tools for *inspection* tasks since they can work tirelessly in complex or hazardous environments and reach high levels of accuracy and performance.

Some of the most propitious scenarios for robot inspection are civil infrastructures, such as bridges, tunnels, roadways, railways, power lines, storage tanks or large facades [16]. These facilities are usually large, complex, constrained or dangerous, so humans cannot inspect them without having adequate resources: e.g., scaffolding, machines, etc. Conversely, robots can cover large areas in reduced times, access to remote and difficult places, and work under harsh conditions and dangerous contexts. Multiple types of robots are used for inspecting civil infrastructures, including not only ground robots [17] or aerial robots [18], but also others capable of crawling and climbing [16].

Moreover, sensors and actuators equipped in these robots can be very diverse, including those visual, strength-based, ultrasonic, magnetic, electrical, thermal, radio waves or endoscopes [17]. In fact, the use of robotic systems for the inspection of structures implies a set of challenges that need multiple sensors, such as coverage for path planning, data gathering or model reconstruction [19]. Inspection of pipes is another important task in the industry. As an example, a ship usually has among 15,000 and 40,000 pipes with different features (e.g., shape, size and material) depending on their use (e.g., fluid, temperature and pressure) [20]. Therefore, the inspection of pipes is a key task in shipyards that need to be performed not only to build but also for maintenance and repairs [21].

A lot of scenarios are especially appropriate for robots, but usually require specific morphologies with special capabilities. As an example, that is the case of offshore oil and gas industries [22] or other high-risk confined spaces [23].

Hyper-redundant robots can contribute not only to improve the performance in some of the reported scenarios but also to others yet unexplored. Due to their large number of degrees of freedom, these robots can operate in constrained environments, reaching unstructured scenarios, navigating in places with a high density of obstacles and operating in narrow spaces like pipes [3]. Additionally, three types of hyper-redundant robots that can be used for inspection purposes: manipulators, mobile manipulators and those with locomotion capabilities. Hyper-redundant manipulators are those constrained to fixed bases [24], whereas hyper-redundant mobile manipulators are integrated with automated and mobile bases, increasing their autonomy and workspace [25]. Finally, hyper-redundant robots can also take advantage of their high flexibility to move and manipulate in complex environments like snakes do [26].

Some of the most relevant hyper-redundant robots intended for inspection purposes have been selected from the scientific literature. OctArm V [27] is a soft robot similar to an elephant trunk installed on a ground robot. It consists of 3 sections actuated by 9 pneumatic muscles and is proposed to perform search and rescue tasks. Woodstock [28] is also a hyper-redundant robot installed on a mobile base and devised for search and rescue tasks. It can be classified as discrete and has 12 degrees of freedom distributed in 6 sections, controlled by 12 distributed motors. OC-Robotics Series II X125 [29] is a very dexterous hyper-redundant manipulator designed for inspection tasks and divided in 12 sections. Tendril [30] is a minimally invasive continuous robot, intended to perform inspection tasks in the space, which has 4 extrinsic motors and 2 sections. Also, a cable-driven hyper-redundant manipulator is developed in [31] with 24 degrees of freedom, 12 sections and 36 motors. The robot that will be presented in this work follows the same structure, provided with 14 degrees of freedom distributed in 7 sections and actuated by 21 motors (Section 3).

Given that these robots can move in the 3D space adopting a wide range of shapes, recent works propose to use immersive technologies to command or supervise their movements. There are multiple devices capable of creating immersive worlds and managing user interactions: the most used for VR are the HTC Vive and Oculus head-mounted devices, whereas the most common in AR are the Microsoft Hololens glasses. Adaptive and immersive interfaces have shown improvements in situational awareness, workload and performance of operators that manage multi-robot systems with both aerial, ground and manipulator robots [32]. Other recent work demonstrated the feasibility of reconstructing scenarios with RGB-D sensors and, at the same time, operating robots through VR head-mounted devices [33]. Others used Augmented Reality to teleoperate an industrial robot for assembly tasks, increasing the accuracy, efficiency and reducing the differences of skillfulness [34]. In fact, multi-target haptic guidance has been studied for a variety of applications, ranging from haptic computer menus to virtual reality applications [35]. Additionally, previous works demonstrated the potential of Mixed Reality in the operation of robots, revealing improvements in situational awareness, workload and efficiency [11,36].

## 3. Robot Design

The robot presented in this work is intended to perform inspection tasks in constrained environments. For that purpose, the design should take into account the following requirements. First, it needs to be hyper-redundant and have many degrees of freedom to increase its dexterity in unstructured scenarios. According to the size and potential applications, diameters from 100 to 120 mm and an extended length in between 700 and 900 mm would be preferable. Additionally, the robot should be modular to allow different lengths and the actuation box should be reusable for different redundant manipulators. At the same time, it needs to be long-lasting, so it should be made by resistant materials. Moreover, the robot should be able to carry payloads of 1 kg to move different sensors for inspection purposes. In addition, lastly, it should have high accuracy, repeatability and resolution.

**Figure 1:**
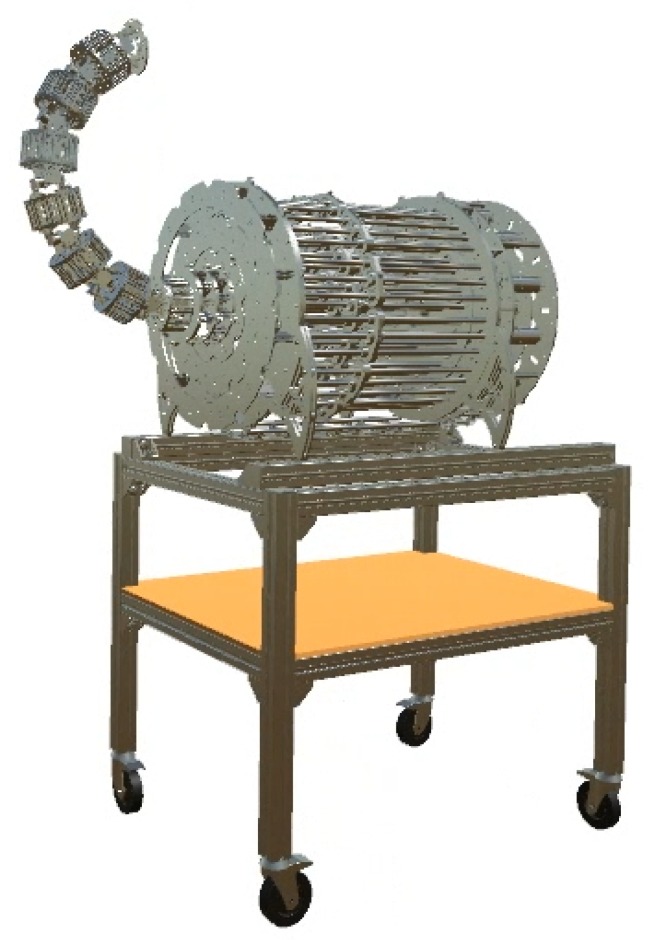
Complete mechanical design of a tendon-driver, discrete and hyper-redundant manipulator with 14 degrees of freedom and 7 sections.

As a result, the robot is designed to be hyper-redundant and discrete. As it will be explained, it has 14 degrees of freedom and 7 distinguishable and rigid sections (Figure 1). Its long structure will be driven by tendons and designed to carry out different applications, inspection tasks included. Following sections will describe the mechanical design (Section 3.1), the actuation system (Section 3.2) and the electronics of the robot (Section 3.3).

### 3.1. Mechanical Design

The design of the robot as a mechanism is constrained by the previously mentioned requirements. Under these circumstances, it is very important to highlight that a good design is the result of a compromise between different requirements that may conflict between each other.

**Figure 2:**
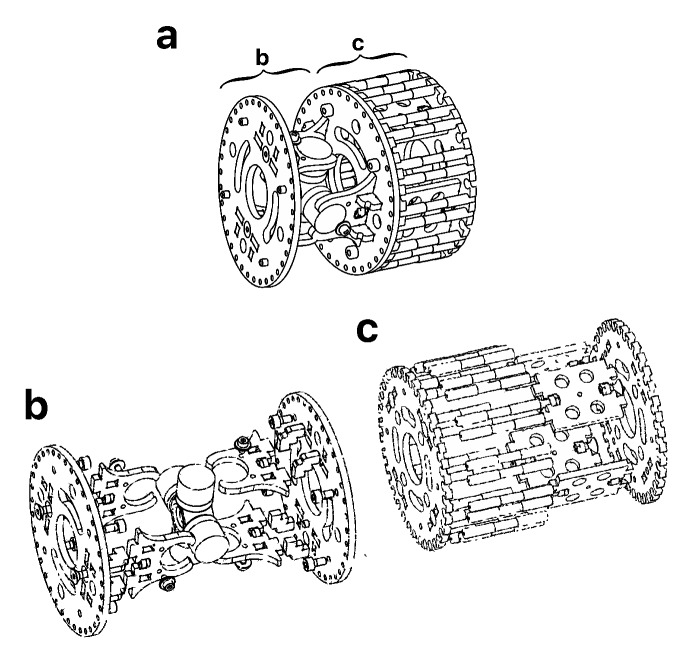
(**a**) Drawing of the base module. (**b**) Exploded view the static segment of the base module devised to increase the robot length. (**c**) Exploded view of the universal joint.

The robot was required to be modular, which usually consist on the repetition of a module in the kinematic chain. Sometimes this module is designed to decrease in size from the base to the tip. However, the robot is less modular and expandable following that scheme. Therefore, the size of the modules was kept constant along the kinematic chain with a diameter of 108 mm (Figure 2a). In every module, the links were extended by appending a rigid section to the joints to increase the total length, which is L=675 mm (Figure 2b). In addition, a set of universal joints were selected to fit the desired size requirements (Figure 2c).

A total of 7 modules were selected as adequate to obtain maximum bending angles of 33° between consecutive sections and an accumulated total bending angle of 230° in the whole body. This parameter is very important in hyper-redundant robots since it is useful to quantify their dexterity. Additionally, the material for the base module needed to be light and resistant, so aluminum was chosen as a good candidate. As a result, every module weighs m=0.681 kg and requires an appropriate actuation system.

### 3.2. Actuation

Depending on the location of the actuators, it is possible to distinguish between *distributed* actuation, when it is located within the robot mechanism itself; or *remote* actuation, in those cases where the movement is transferred from an external location.

Robots devised for inspection tasks can be very different in size depending on each application. Distributed actuation systems are not possible to be reused for robots with different sizes. Additionally, that setup would highly increase the total mass of the robot and, consequently, the power and the size of the actuators as well. Thus, the actuation system was chosen to be remote from an external actuation box based on DC motors and twisted steel cables.

**Figure 3:**
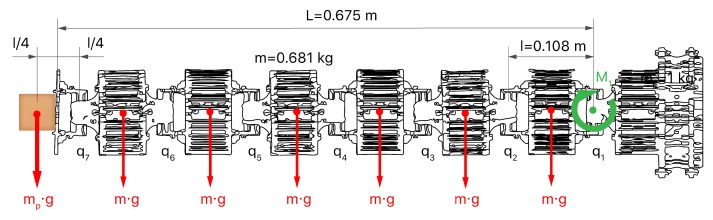
Free body diagram of the required torque exerted by the first joint to balance a payload and the weight of the robot body.

Based on this type of actuation, it is possible to calculate the nominal torque needed for the presented robot. Static forces were only taken into account since dynamic ones can be neglected when the robot performs with high cycle times. Figure 3 depicts a free body diagram of the robot body with a straight configuration, perpendicular to the action of gravity. Theoretically, this configuration is the most disadvantageous pose for the robot in terms of load. The torque ($M_{1}$) will find its maximum value in the first joint ($q_{1}$), as a result of the influence of gravity (*g*) in the mass of every module (*m*) multiplied by their distance to the first joint ($l_{i}$). Also, a payload of mass $m_{p}$ was included according to the robot requirements, located in the endpoint at a distance of $l_{p}$ from the first joint (Equation (1)).

(1) $$M_{1}=\sum_{i=1}^{7}\left(m\cdot g\cdot l_{i}\right)+\left(m_{p}\cdot g\cdot l_{p}\right)=11.919+6.886=18.8N\cdot m$$

**Figure 4:**
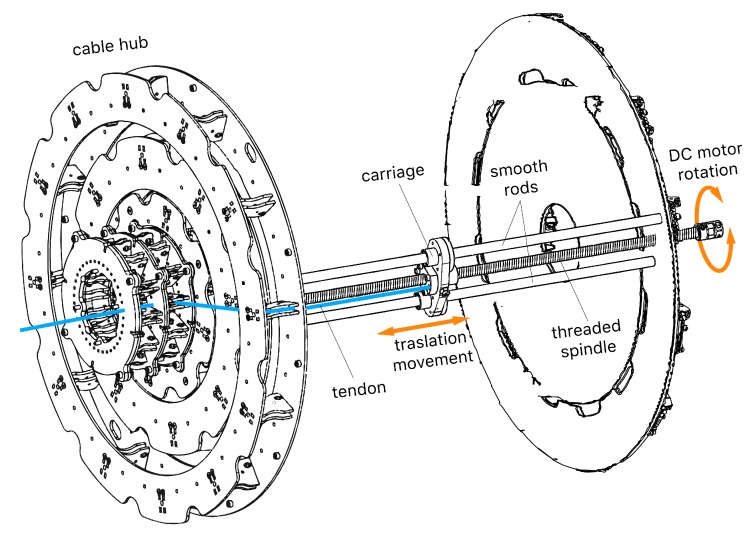
Drawing of the actuation system for just one tendon. The DC motor is connected to a threaded spindle that converts the rotation to a linear movement of a carriage, in which a tendon is anchored and then guided through a set of pulleys to the robot.

Therefore, the selected DC motors are required to have enough power to balance the torque generated by the weight of the robot and the payload. A factor of safety is applied in numerous steps of the design, this one included. Also, it is important to select the motors depending on their speed. In this case, the torque generated by each motor is transferred to a threaded spindle that converts the rotation to a linear movement. Then, a carriage with internal threading and bearings is screwed in the spindle and fixed to 2 smooth rods to obtain the linear translation. In fact, this system is a mechanical speed reducer that will determine the resolution, accuracy and speed of the robot. Following this design, the spindles and rods needed to have a minimum length equal to the maximum displacement of the tendons (Figure 4).

**Figure 5:**
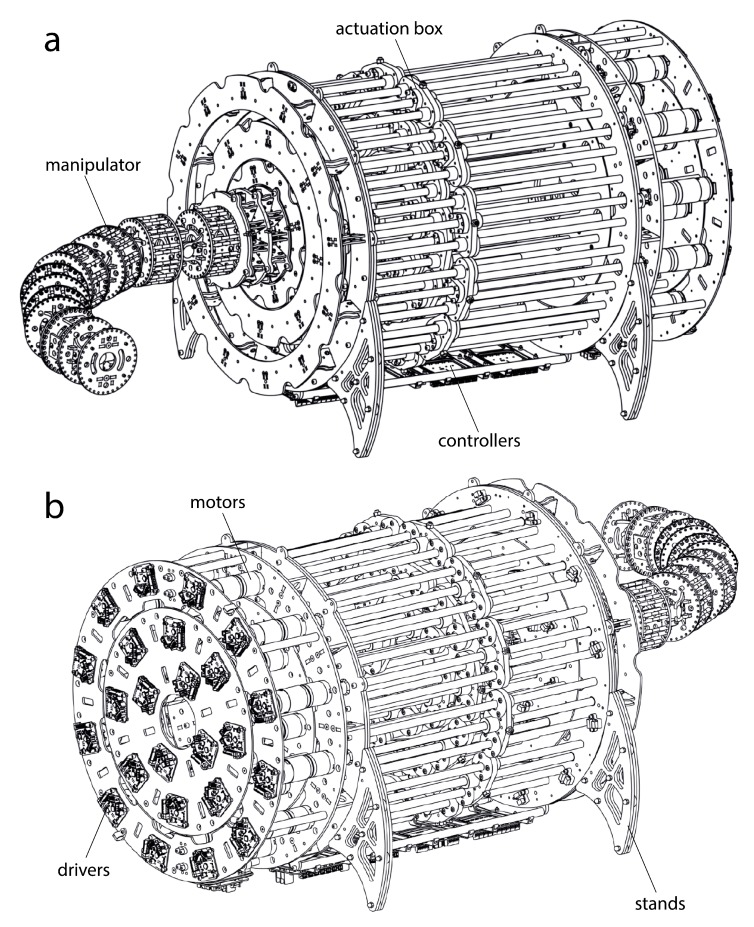
Perspective (**a**) front and (**b**) back view of the complete design of the hyper-redundant robot.

The resulting mechanical design is very sturdy and durable. The final mechanical model can be seen in Figure 5. It has been designed to have a high resolution, accuracy and moderately high speeds for inspection purposes.

### 3.3. Electronic Design

**Figure 6:**
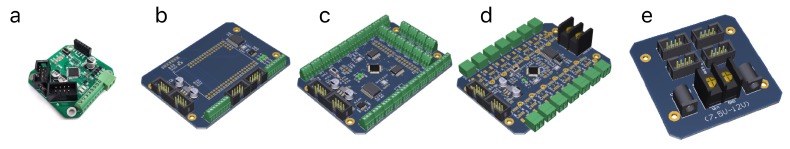
Customized design of different electronic boards. (**a**) Motor drivers for a closed-loop control using encoders. (**b**) Electronic board to supervise the limit switches. (**c**) Central controller to manage all the communications. (**d**) 24 *V* power board to supply the motors. (**e**) 9 *V* power board to supply energy to the rest of electronic boards.

The lack of commercial solutions with high standards of synchronization and communication capacity leads to the customization of the electronic system for this particular robot. First, each driver of the robot is designed to obtain a closed-loop system using the feedback from an encoder (Figure 6a). All of them are devised to allow communications via Controller Area Network (CAN) bus to simplify the wiring process. Then, other 4 additional circuits are manufactured: one board to communicate the state of the limit switches (Figure 6b), a central controller to manage all the communications (Figure 6c), a 24 *V* power board (Figure 6d) and a 9 *V* power board (Figure 6e).

## 4. Kinematic Control

Once the robot is fully designed and built, it is time to study how to manage its movements. For that purpose, it is possible to distinguish three different types of control. First, low-level control regulates electrical inputs to obtain a desired output in the actuators (Section 4.1). Then, mid-level control supervises how to transform the values of the actuators to rotations in the joints (Section 4.2). Finally, high-level control is proposed to manage the rotations of the joints to useful movements for the whole kinematic chain (Section 4.3).

### 4.1. Actuation Control

The robot is actuated by 21 extrinsic motors, but the actuation box is expandable to use 3 additional ones for end-effector tools. Each of them has an encoder that is used for feedback and regulated by drivers (Figure 6a).

**Figure 7:**
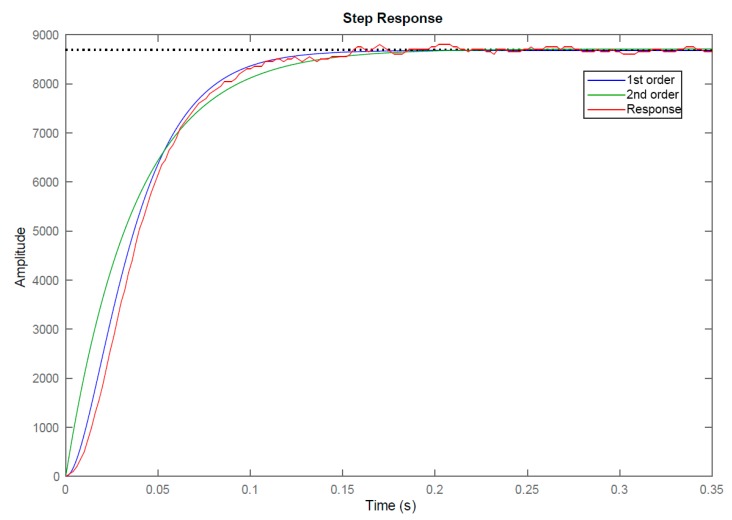
Step response for the motor identification fits to a first or underdamped second-order system.

The initial step to control the motors is the *identification*. It helps to understand its behavior against variations in the inputs to adjust the outputs as desired. For that purpose, a motor load was applied to analyze a step response and obtain the correspondent transfer function (Equation (2)). As a result, it was analyzed and could be either approximated by a first or a second-order function. An underdamped second-order response was finally chosen to provide a better fit (Figure 7).

(2) $$G\left(s\right)=\frac{95900}{\left(s+65.48)(s+43.02\right)}$$

**Figure 8:**
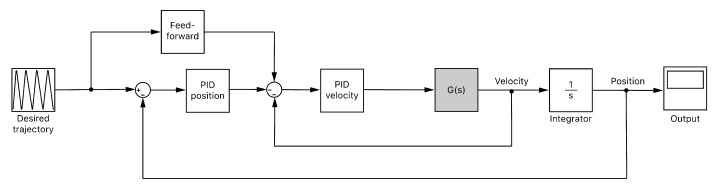
Control scheme for the motors. Two feedback controllers are used both for velocity and position and a feed-forward scheme is implemented to correct undesirable delays in the response.

Once the behavior of the motor is modelled, a closed-loop control scheme is developed (Figure 8). The input of the system is the desired position trajectory for each motor. Such input will be previously limited in velocity and acceleration according to the functioning capabilities of the motors. The output of the system will be the motor velocity that leads to the desired position. Two Proportional-Integral-Derivative (PID) controllers are employed to close the loop: one for the velocity and another for the position. Additionally, a feed-forward control is applied to the input of the PID controller for the velocity, looking for avoiding undesirable delays in the response. This PID was tuned and provided with an anti-windup filter. On the other hand, the PID controller for the position was simpler since the integral and derivative actions could be neglected.

### 4.2. Joint Control

**Figure 9:**
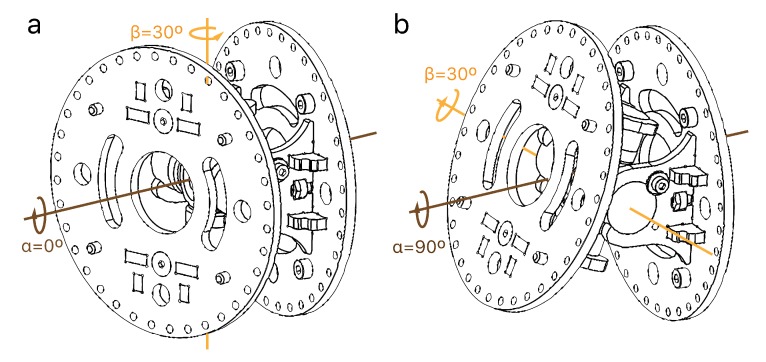
The robot is comprised by universal joints, each one with 2 degrees of freedom. The rotations are defined by two angles α and β which do not directly represent the rotation axes, but the direction and the magnitude of the rotation, respectively. (**a**) Rotation around one axis. (**b**) Rotation around the perpendicular axis.

Once the motors are appropriately controlled, it is possible to obtain a reliable function that relates the linear displacements of cables (Figure 4) to the rotations of joints. First, the robot is calibrated in a zero-angle pose and then, a reference system is created to measure the rotations and displacements. Two angles define each joint rotation: α represents the direction to rotate the joint, whereas β is dependent on α and represents the magnitude of the rotation (Figure 9). Thus, the resulting rotation (*R*) could be defined using the Rodrigues’ rotation formula (Equation (3)), where *I* is the identity matrix and *K* the cross-product matrix (Equation (4)).

(3) $$R\left(\alpha ,\beta \right)=I+sin\left(\beta \right)K\alpha +\left(1-cos\left(\beta \right)\right){\left(K\alpha \right)}^{2}$$

(4) $$K=\left[\begin{array}{ccc} 0 & -k_{z} & k_{y}\\ k_{z} & 0 & -k_{x}\\ -k_{y} & k_{x} & 0 \end{array}\right]\mathrm{where}\mathrm{for}\mathrm{this}\mathrm{robot}:\left[k_{x},k_{y},k_{z}\right]=\left[cos\left(90^{\circ }+\alpha \right),sin\left(90^{\circ }+\alpha \right),0\right]$$

**Figure 10:**
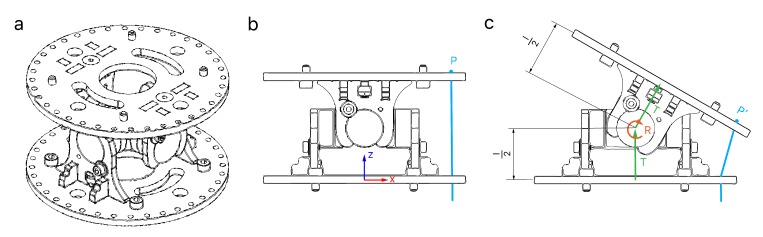
(**a**) Obtaining the displacements of the cables for a single joint. (**b**,**c**) Transformation of the pull point *P* to $P^{\prime }$ after a joint rotation.

Each module is driven using 3 equidistant cables at 120° from each other. The displacements of each cable when the joint rotates can be obtained applying homogeneous matrix transformations (Equation (5)). The position of each cable *P* after a rotation will lead to $P^{\prime }$ applying a translation, *T* (Equation (6)), then the desired rotation, *R* (Equation (3)), and finally the same translation again, *T*, as depicted in Figure 10.

(5) $$P^{\prime }=TRTP$$

(6) $$T=\left[\begin{array}{cccc} 1 & 0 & 0 & 0\\ 0 & 1 & 0 & 0\\ 0 & 0 & 1 & l/2\\ 0 & 0 & 0 & 1 \end{array}\right]$$

**Figure 11:**
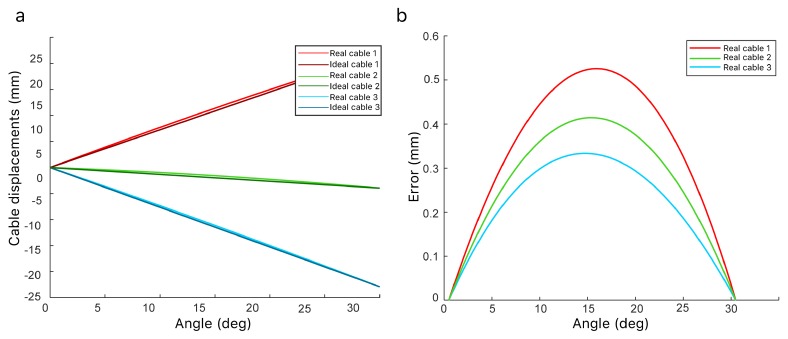
(**a**) Difference between the real displacements of the cables of the same module versus the angle magnitude. (**b**) Error between the real displacements and the linear assumption.

It is important to highlight that theoretically, the relation between the angle β and the increment in the length of the cables is not perfectly linear (Figure 11a). However, the linear model will be used because the errors obtained from this assumption are negligible (Figure 11b).

Finally, trapezoidal speed-time curves were managed to generate coordinated trajectories. For this purpose, acceleration and deceleration speeds are established as well as the maximum global speed (0–8000 pulses-per-second). Then, the speed of all the motors is intentionally slowed down, so their movements begin and end in sync with the motor that needs to rotate the most.

### 4.3. Kinematic Chain Control

Once the control of joints is adequately managed, high-level controls are intended to manage the movements of the whole robot body of the robot. Thus, such strategies will transform the independent joint movements in global ones for the kinematic chain to perform specific applications.

It is possible to cite some of the most common control practices in this field. For example, the inverse kinematics aims to find the joint values that lead to a position and orientation for the end-effector. This problem is relatively easy to be solved for non-redundant robots. However, hyper-redundant ones offer a set of infinite possible solutions for each location of the end-effector. Therefore, selecting the most adequate is not always a simple task.

However, methods aiming to solve the inverse kinematics problem do not usually exploit all the kinematic capabilities for such robots. Thus, the shape control appears to manage the whole configuration of the robot relying on a multi-variable reference. The main challenge for this control is finding a good method to efficiently command such references in the three-dimensional space. Traditional methods were complex to design or not fully adapted to the needs of this kind of robots. Fortunately, immersive technologies emerge as the best solution to teleoperate hyper-redundant robots for teleoperation applications [11].

Another remarkable high-level control is the follow-the-leader. It is oriented to introduce hyper-redundant robots in constrained environments, so their body follows the path travelled by the tip. Multiple other strategies are usually created ad-hoc for each specific application. However, recent work raises an analysis of multiple behaviors, intrinsic to the kinematics of hyper-redundant robots that may unify a framework for multiple high-level controls [37].

This work will use the Natural-CCD algorithm to control the presented robot since it is based on simple principles and provide accurate solutions while being computationally efficient for real-time applications. Additionally, it allows multiple customization options, manages restrictions and collisions and provides intermediate configurations to reach the final solution [7]. It relies on an iterative approach that uses small increments to control the movement of each module of the robot leading to very simple and fast-converging solutions. As a result, it provides multiple high-level controls such as those previously mentioned for the inverse kinematics problem, shape control or follow-the-leader techniques, among others. Section 5 will use this algorithm in conjunction with a Mixed Reality environment to perform a specific task based on inspection.

## 5. Teleoperation and Mixed Reality

The first industrial revolution used water and steam to mechanize production, the second used electric energy to create mass production and the third used electronics and information technology to automate production. Industry 4.0 is the next paradigm shift, and it does not aim to produce more but to customize and optimize the production process. Robotics and immersive realities are considered to be some of the most important technologies to achieve that goal [38]. In fact, inspection tasks are very common in the industry for maintenance, supervision and evaluation of machines, processes and infrastructures, among others.

This section will present an analysis of an inspection task in a real environment using such technologies. Specifically, it consists on the inspection of a boiler room looking for anomalies. These environments can be hazardous and very unstructured with complex pipe structures, so using dexterous robots can be a good idea without the presence of human beings. However, these tasks usually involve high risks and sometimes require the taking of unexpected and complex decisions. Thus, human intervention is usually desirable from remote and safe locations. Hyper-redundant robots are devised for this kind of tasks. Additionally, recent work claims that immersive technologies can be the best solution to ease their spatial understanding and command their shape in the three-dimensional space [11].

**Figure 12:**
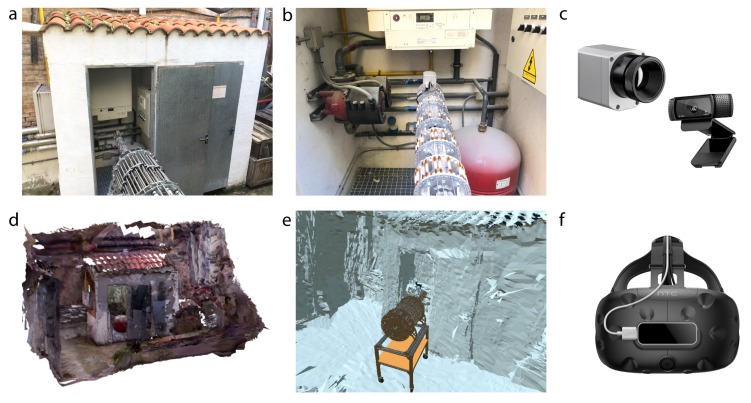
(**a**) Boiler room with the robot prepared to perform an inspection. (**b**) First-person view of the robot and the thermal camera located in the end-effector. (**c**) Two sensors were used in the real environment: an Optrix PI thermal camera and a Logitech RGB camera. (**d**) On the other hand, the real scenario was reconstructed using a Kinect and the software RTAB-Map. (**e**) Then, it was imported in Virtual Reality with a model of the robot. (**f**) The operator uses the HTC Vive VR headset and the Leap Motion controller to teleoperate the real robot from a remote and safe location.

As a result, the system setup is comprised of two workspaces: the robot is in the boiler room while an operator safely commands its movements from a safe and remote location. In the boiler room (Figure 12a), the robot is disposed to perform the inspection task with 2 on-board sensors: an RGB-color camera and a thermal camera located on its end-effector (Figure 12c). On the other hand, an operator is in a remote location, provided with a Head-Mounted Display (HMD) to command the robot movements using Mixed Reality. Specifically, the operator will be introduced in a virtual environment previously reconstructed in 3D using structured-light techniques (Figure 12d). This virtual scenario was recreated mimicking the real one to increase the situational awareness of the operator. The HMD used is the HTC Vive headset in conjunction with the Leap Motion controller to track the hand movements (Figure 12f). As a result, the operator has visual, aural and vestibular feedback to effectively command the robot movements. Making an appropriate selection of the sensors in both spaces is crucial to obtain a good teleoperation performance.

Also, communications and delay management are very important since they determine if the system is suited for real-time applications. Thus, a bilateral architecture was implemented using sockets and a TCP/IP connection. Furthermore, the designed teleoperation follows a position–position scheme. In other words, the robot will move to a determined position previously commanded by the user. Although this scheme is very simple, it is the best suited for this application, since the size relation of the master-slave is similar, there are small limitations in the slave speed, a high level of telepresence is required, and the task involves a high number of DoF [39].

**Figure 13:**
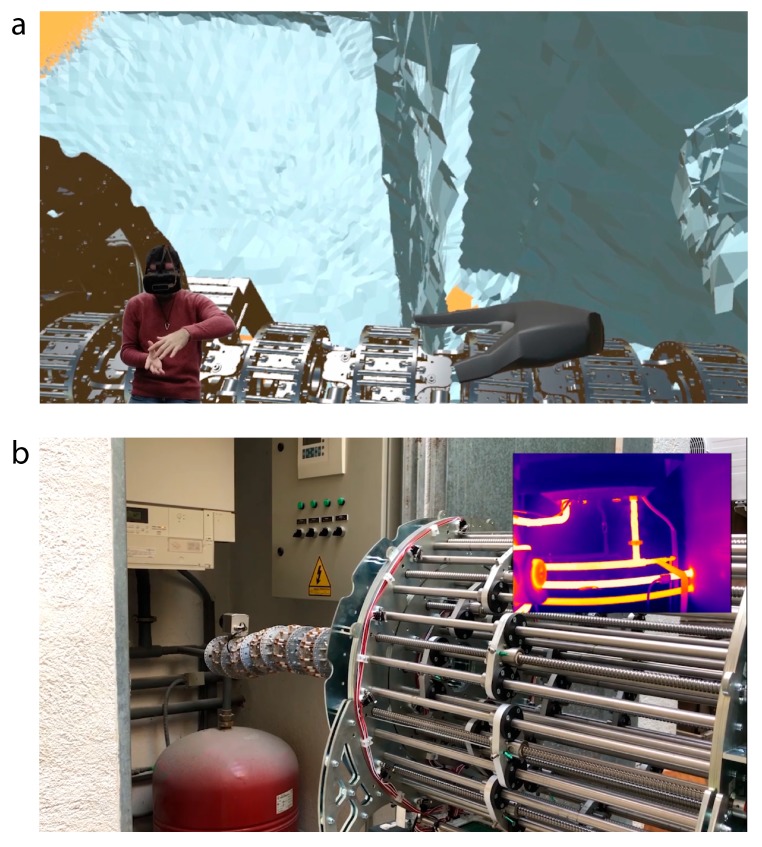
(**a**) The operator performs a shape control using his bare hands to interact with the virtual robot, a replica of the real one with the same kinematics and physical restrictions. (**b**) Using Mixed Reality, the real robot (slave) is moved to adopt the same pose as the virtual one (master). The thermal camera located in the end-effector let the operator supervise the temperatures of the boiler room from a safe and remote location.

In summary, the operator will use Mixed Reality to move an exact replica of the robot in the virtual environment. The user will be able to use the bare hands to interact with the virtual robot and command the real one using shape-control techniques. These hands are tracked and displayed in the Virtual world and are provided by a collision model, as well as the robot. When both enter in contact, the robot pose moves without opposing force, complying towards the desired shape. The resulting telepresence is very high given that the robot size, movements, restrictions and its environment in the virtual world match with the ones in the real world (Figure 13a). Then, once the desired reference is ready, the operator pushes a virtual button and the signal is sent to the real one. As a result, the real robot moves accordingly to the configuration commanded in the virtual world. With this strategy, the user is safe from a remote distance while at the same time, has the perception of being in the boiler room. Simultaneously, a video streaming with the output from the thermal camera allows supervision of any abnormal temperatures inside the boiler room (Figure 13b). Thanks to the high versatility of the hyper-redundant robot, it can orient the end-effector with high dexterity whereas a traditional, non-redundant one would have struggled to perform the same task. The accuracy of the robot was found to be below 1 millimeter on average and the control errors from a high-level perspective converged to 0.1% relative to the total length of the robot in both the inverse kinematics and the shape control. These standards are much higher than those needed to complete the inspection task, so the robot is expected to outperform in this scenario.

As a result of the inspection and using the RGB camera, it is concluded that there is no appreciable damage, but just some debris that may cause malfunction problems in the pumps in the long-term period. On the other hand, the hyper-redundant robot allowed to inspect the constrained space using the thermal camera. The pipe and pump temperature was adequate and homogeneous, which implies that the boiler is performing appropriately. However, the heat seemed to undesirably expand by conduction through small metal elements and the room walls deteriorating the infrastructure. Thus, thermal insulation was recommended to be applied in certain locations to prevent future damages. An explanatory video of these experiments can be found in Supplementary Material.

## 6. Conclusions

This work introduces the design process, kinematic control and teleoperation using Mixed Reality of a discrete hyper-redundant robot for inspection tasks. For the design process, it can be concluded that a good solution is the result of a compromise between different requirements that may conflict between each other.

For example, a good hyper-redundant robot for inspection tasks would be modular, lightweight, robust, accurate, with low cycle times, high repeatability, high resolution and high maximum bending angles to grant great dexterity. However, these characteristics should be adequately tuned depending on each application since current materials and technologies do not allow the construction of an ideal hyper-redundant robot.

Moreover, a distributed location scheme of the actuators throughout the robot body was found unfeasible, unlike for traditional robots. It is an unsustainable design strategy because hyper-redundant robots have a lot of degrees of freedom, hence a lot of actuators. This large number of actuators in the robot body adds more weight to the structure, requiring more power to balance this extra-weight, forcing to use even bigger actuators, and creating a vicious circle.

Thus, a remote actuation scheme was selected to allow a lighter design with further minimization possibilities. Related to this, choosing the right materials is also crucial to get the optimal dimensions. Flexible materials would lead to continuous or soft robots, instead of rigid ones, increasing the compliance but decreasing the payload or the robustness. Consequently, finding the right design will be highly dependent on the application and the environment.

The presented robot was also controlled from the actuators to high-level strategies based on the management of the whole kinematic chain. It is important to highlight that a kinematic control is reliable enough since dynamic forces can be neglected when the robot performs with moderately high cycle times. In contrast to traditional robotics with the actuators distributed in the robot body, the selected remote actuation scheme forces to calculate an intermediate control step to link the output of the actuators to the joints. Relating to the latter, it is very important to appropriately choose the actuators as well as the speed reducer system because they will highly determine the dimensioning, payload, resolution, accuracy and speed of the robot.

Additionally, the designed robot was tested in a real environment within the framework of Industry 4.0. Specifically, it was teleoperated to conduct an inspection task in a boiler room using Mixed Reality in real time. These practices are very useful when the real environment is potentially hazardous or adverse. Both the boiler room and the robot were replicated in a virtual world, where the user could use the bare hands to interact with the virtual robot and command the movements of the real one. Many sensors were used for this application from encoders, thermal and RGB cameras, a head-mounted display with 6 DoF or a hand-tracking visual system. Making an appropriate selection of these sensors is crucial to obtain an optimal teleoperation performance. Consequently, the operators experienced high situational awareness and performed with high efficiency from a remote and safe location. Results from the inspection task revealed that the boiler was functioning adequately whereas application of thermal insulation was recommended in certain locations to prevent future damages.

The initial hypothesis of this work stated that hyper-redundant robots, like the one presented, can be a good solution to perform complex tasks in remote environments. The experimentation process in both simulated and real environments validate this assumption. It cannot be neglected that further work needs to be acknowledged, especially in terms of design optimization, finding the right balance within the robot requirements and relying on each application. However, there are a lot of potential using this kind of robots for inspection purposes. Selecting the robot as hyper-redundant was definitely a good solution to inspect unstructured and constrained spaces, like the one presented. Moreover, it was found that immersive technologies are an efficient alternative that let humans teleoperate robots from remote and safe locations with great situational awareness. Thus, it can be concluded that these technologies, robotics and immersive realities, should play an important role in the near future of automated and remote applications, especially with the upcoming paradigm shift of Industry 4.0.

## Supplementary Materials

Electronic Supplementary Material: An explanatory video of this work can be found in https://www.mdpi.com/1424-8220/20/8/2181/s1.

## Abbreviations

The following abbreviations are used in this manuscript:

3D	Three Dimensions
DoF	Degree-of-Freedom
VR	Virtual Reality
AR	Augmented Reality
MR	Mixed Reality
CAN	Controller Area Network
PID	Proportional, Integral, Derivative
HMD	Head-Mounted Display
